# Effects of Six Weeks of Flywheel Single-Leg Romanian Deadlift Training on Speed, Jumping and Change of Direction Performance

**DOI:** 10.3390/ijerph19031200

**Published:** 2022-01-21

**Authors:** Esther Morencos, Pablo González-Frutos, Carlos Rivera, Santiago Veiga

**Affiliations:** 1 Faculty of Health Sciences, Universidad Francisco de Vitoria, 28223 Madrid, Spain; esther.morencos@ufv.es (E.M.); carlos.rivera@ufv.es (C.R.); 2 Sports Department, Faculty of Physical Activity and Sport Sciences, Universidad Politécnica de Madrid, 28040 Madrid, Spain; santiago.veiga@upm.es

**Keywords:** performance, eccentric training, countermovement jump, change of direction, sprint

## Abstract

Several studies have confirmed the efficacy of flywheel training, mainly in the bilateral half-squat exercise. The aim of the present study was to analyze the effects of single-leg Romanian deadlift flywheel training on speed, jumping and change of direction performance. Seventeen young healthy males underwent two periods of 3-week training based on two weekly sessions of 3 sets × 7 repetitions or 4 sets × 7 repetitions of single-leg Romanian deadlifts (0.037 kg/m² moment inertia) with their dominant and non-dominant leg. After the first three weeks of the program, the CMJ, the 10 m, 30 m and total sprint times, as well as the COD-90 test, presented likely substantial beneficial effects and a small decrease in the relationship between the sprint and COD-90 test performance. After the second period of the three-week training, likely detrimental effects were observed in some of the change of direction conditions and an increase in the relationship between the sprint and the COD-180 performance. It could be hypothesized that most of the flywheel training effects reported in the traditional protocols lasting a minimum of 5–6 weeks would occur in the first weeks of training.

## 1. Introduction

Different training devices, such as flywheel(s), have been developed and extensively used in recent years to improve several aspects of strength and power related to sport performance [1]. These devices are designed to provide resistance by the torque generated by rotating flywheels during acceleration (concentric) and breaking (eccentric) movement phases. Both eccentric and concentric strength are key components in almost any sport activity (i.e., sprint acceleration, jumping, change of direction, etc.) [2], especially those that require the generation of force over a short period of time [3]. The ability of rapidly breaking the movement enhances the amount of elastic energy stored in the muscular groups, optimizing the stretch–shortening cycle and, therefore, contributing to increased acceleration during the concentric movement [4].

Several studies have confirmed the efficacy of flywheel training devices for improving hypertrophy [5], power [6], countermovement jump (CMJ) performance [4,7,8], 10 m sprint time and changes of direction (COD) [4,7,8,9], and injury prevention or rehabilitation [4,10,11]. Most studies agree that familiarization processes, adequate progression, and load control (encoder) are required. However, to the best of our best knowledge, there is no research that analyzes load progression in the methodology. Although there is some approach in the literature to establish guidelines [12,13], there is still a lack of specific information that makes it difficult to prescribe and control training programs using flywheel devices.

Previous findings on flywheel training suggest that unilateral training is more effective in improving COD-90 performance, through a comparison of squat and lunge exercises [14]. In this study, participants performed a 6-week program, 2 days/week, 4 × 7 (set × reps) with 3 min recovery between sets, using 0.05 kg/m^2^ moment inertia. Another study recommended bilateral half-squats to improve COD tasks, whereas a one-leg half-squat would be the best option to improve the horizontal jump ability [15]. Training prescription consisted of 6 weeks, 2 days/week, 4 × 8 (set × reps) with 2 min recovery between sets, using 0.025 kg·m^2^ moment inertia. Both studies performed knee-dominant exercises; however, hamstring injuries are some of the most common musculoskeletal injuries in competitive sports [16], due to their crucial role in producing propulsive force during acceleration [17]. Therefore, unilateral lower-body movement, such as the single-leg Romanian deadlift, employed to build the stability, strength, and power of the athletes’ posterior chain and core [18], could be employed to examine the training effects using flywheel. 

Furthermore, more research is needed to apply the wide variety of intervention protocols in practice [19]. Due to the diversity in study designs, and varieties in the kind of equipment—cylindrical shaft, conical pulley, etc., based on single or multi-joint exercises and different moment inertia [1,20]—more evidence-based information is needed for its wider implementation in daily practice. Therefore, this study aimed to develop the knowledge of flywheel training, analyzing the effects of the single-leg Romanian deadlift using a flywheel device on physical performance (measured as lower limb power, 30 m sprint (10–20–30), CMJ, COD-90 and COD-180). To analyze the adaptation and progression of load, these tests were measured before (PRE), and after 1 (post-1) and 2 (post-2) three-week periods. 

## 2. Materials and Methods

### 2.1. Participants 

Seventeen young, healthy, male sport science students were included in the study (age: 22.8 ± 2.5 years; body mass: 75.2 ± 7.9 kg; height: 175.6 ± 5.4 cm). All subjects had been actively involved in strength training (at least twice a week) during the last three years and were familiar with execution of the single-leg Romanian deadlift technique, although they were novices in structured flywheel training. The experimental protocol was fully explained to all participants, and they gave written consent, in accordance with the Declaration of Helsinki II. The Local University Ethics Committee approved the study.

### 2.2. Experimental Procedures

**Figure 1:**
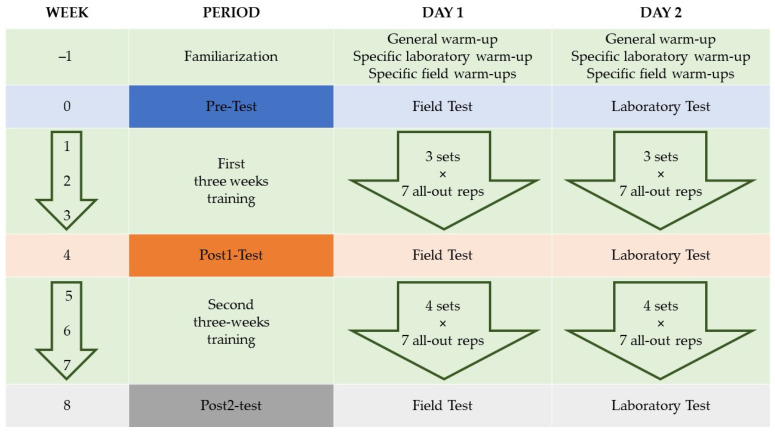
Experimental procedures.

**Figure 2:**
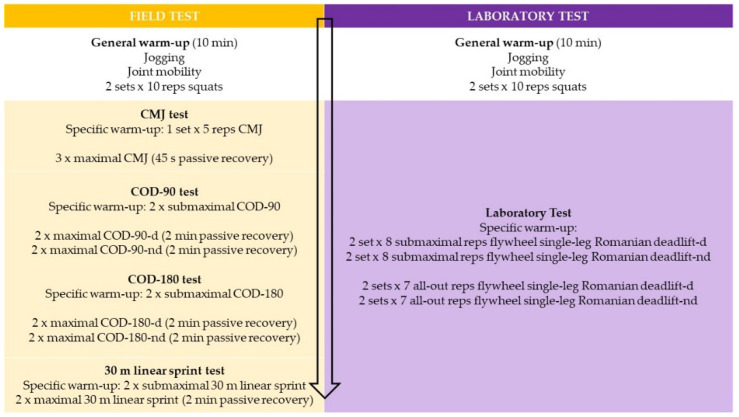
Field and laboratory testing protocols employed to evaluate effects of flywheel training.

Laboratory and field tests were measured before (pre), and after 1 (post-1) and 2 (post-2) periods of three weeks of single-leg Romanian deadlift training using a flywheel machine (Figure 1). Field tests were performed on an indoor futsal court in the following order: CMJ, COD tests, then the 30 m sprint. Lower limb power was assessed using a flywheel device in the laboratory, seventy-two hours after the field tests (Figure 2). Participants were asked to follow a similar diet compared with the days prior to the experiment and to abstain from performing any strenuous exercises on the day before each test. In addition, they were allowed to complete two familiarization sessions with specific field and laboratory test warm-ups to control for any learning effects. 

### 2.3. Field Tests

After a 10 min standardized general warm-up, which included jogging, joint mobility exercises, and 2 sets of 10 repetitions of squats, field tests were carried out. Additionally, a specific warm-up was performed before each test: 1 set of 5 reps (CMJ) and 2 activities of submaximal effort (COD and linear sprint) (Figure 2). 

Countermovement jump tests (CMJs) were performed using a contact platform (Chronojump-BoscoSystem, Barcelona, Spain). Jumping height was determined based on the flight time (Jump height = Flight Time^2^ × 9.81/8) with Chronojump software (Chronojump-BoscoSystem, Barcelona, Spain). During the CMJ, all subjects were instructed to place their hands on their hips, which was followed by a vertical jump performed with maximal effort and landing in an upright position, keeping the knees extended. The CMJ was performed three times, with 45 s of passive recovery between them, and the best jump performance was used for further analysis. 

Subjects performed a 30 m linear sprint test, and the time was recorded using photoelectric cells (Racetime2, Microgate, Bolzano, Italy) which were adjusted according to the height of the participants (130 and 150 cm above the floor) [21] in 10 m, 20 m and 30 m line. As with the COD test, the front foot was placed 0.5 m before the first timing gate, and subjects started voluntarily so the reaction time was eliminated. After a specific warm-up, including 2 activities of submaximal effort, two trials were completed. Two minutes of passive recovery occurred between the trials [22]. The best performance for the total sprint time in 30 m (TT-30 m) was used for further analysis, calculating the time taken for each 10 m: 0 to 10 m (10 m), 10 to 20 m (20 m) and 20 to 30 m (30 m).

COD capacity was measured using the protocols described by previous studies [14]. Participants performed four sets of maximum 5 m + 5 m straight line sprints, two with a turn of 90° (COD-90) and two with a turn of 180° (COD-180). In the COD-90 test, the turning space was determined by 5 sticks (height: 1.2 m) which were placed vertically in order to avoid a curvilinear trajectory. One repetition was performed with the dominant leg on the outside (COD-90-d) during the turn, and the other with the non-dominant leg (COD-90-nd). In the COD-180 test, subjects performed a turn by touching a line with their dominant leg (COD-180-d), and then a turn with their non-dominant leg (COD-180-nd). Subjects performed two trials of each test as fast as possible. Two minutes of passive recovery were established between trials and tests. The time was recorded with the same photoelectric cells (Racetime2, Microgate1, Bolzano, Italy), using two of them in the COD-90-d and COD-90-nd tests (placed on the start and finish lines), but only one, placed on the start/finish line, in the COD-180-d and COD-180-nd tests. The front foot was placed 0.5 m behind the first timing gate, and players started voluntarily. The best time of each test (COD-90-d, COD-90-nd, COD-180-d, and COD-180-nd) and the mean values of both legs (COD-90-dnd and COD-180-dnd) were compared with the 10 m times (to calculate the percentage mean speed loss due to executing the COD (DEC-COD-90-d, DEC-COD-90-nd, DEC-COD-90-dnd, DEC-COD-180-d, DEC-COD-180-nd, and DEC-COD-180-dnd), through the formula [(COD − T10 m)/T10 m) × 100], as previously detailed [14].

### 2.4. Laboratory Test

After a 10 min standardized general warm-up, which included jogging, joint mobility exercises, and 2 sets of 10 repetitions of squats, a laboratory test was carried out (power test). A specific warm-up was performed with the same flywheel device: two submaximal sets of 8 reps of single-leg Romanian deadlift exercise (0.037 kg/m² moment inertia) (Figure 2). 

The tests consisted of an assessment of the power in the single-leg Romanian deadlift exercise using a flywheel machine (RSP Squat, RSP Inertial Performance, Pontevedra, Spain) connected to the athlete with an adjustable harness (RSP harness, RSP Inercial Performance S.L., Pontevedra, Spain), allowing subjects to perform maximal CON and ECC actions. Power was sampled at 64 pulses/rpm using a rotatory encoder (RSP-e, RSP Inercial Performance S.L., Pontevedra, Spain) and associated software (RSP app 2.1.1, RSP Inercial Performance S.L., Pontevedra, Spain). Subjects executed 2 sets of 7 all-out repetitions (preceded by 3 submaximal repetitions) of single-leg Romanian deadlifts (0.037 kg/m² moment inertia) with their dominant (Power d) and non-dominant legs (Power nd). The moment of inertia employed was lower than that in previous research [9], to elicit high concentric peak power output values [23]. The best of the two sets, according to the criteria of higher rotational mean power relative to body weight, was considered for subsequent analysis. Participants were instructed to extend their hip as quickly as possible during the work sets. The range of motion was approximately 90°. The participants were requested to push with maximal effort during the entire range of motion in the CON action (from ~90° to almost-full extension), where the strap about the flywheel shaft was rolled out. Then, as the strap rewound, they tried to resist the flywheel force gently during the first third of the ECC action, and then applied maximal effort to stop the movement at about a 90° hip flexion. A 2 min break occurred between consecutive sets, as previously employed [24]. Real-time performance feedback on peak power was always offered to the participants. Training was conducted by the same 3 researchers. The mean power was measured for every coupled CON–ECC repetition [25], calculating the means of 7 all-out repetitions for the following variables: power of the concentric phase with the dominant leg (Power d-con) and the non-dominant leg (Power nd-con); power of the eccentric phase with the dominant leg (Power d-ecc) and the non-dominant leg (Power nd-ecc); average power of the concentric and eccentric phases with the dominant leg (Power d) and the non-dominant leg (Power nd); and average power of both phases and both legs (Power dnd). 

### 2.5. Flywheel Resistance Exercise Training 

Participants underwent two periods of a 3-week training program based on the single-leg Romanian deadlift with the same flywheel device and conditions used in the power test, performing 2 sessions/week with at least 48 h of rest between the sessions. This allowed the participants to overcome most changes in contractile muscle properties after flywheel training that occur within 24 h after exercise [26]. Each session was structured with a brief standardized warm-up (similarly to that used before in the power test) and 3 sets (during the first three-week period) or 4 sets (during the second three-week period) of 7 all-out repetitions (preceded by 3 submaximal repetitions) of single-leg Romanian deadlifts (0.037 kg/m² moment inertia) with the dominant and non-dominant leg. Training was conducted by the same three researchers and the dominant leg order was randomized to control for any bias during the training process. A 2 min recovery period was allowed between sets, and real-time performance feedback of peak power was always offered to the participants. 

### 2.6. Statistical Analysis

First, the Shapiro–Wilk test was selected to confirm normal distribution of data. Then, magnitude-based inferences, effect sizes (ESs) with 90% confidence intervals (CIs) and smallest worthwhile changes (%) between pre- and post-1 and post-2 conditions were calculated using a spreadsheet created for this purpose [27]. ES magnitudes were interpreted as trivial (ES < 0.20), small (ES = 0.20–0.59), moderate (ES = 0.60–1.19), large (ES = 1.20–2.00), and very large (ES > 2.00). Differences were defined as unclear when the confidence limits for the effect size included both substantial positive and negative values (±0.2 × standardization). Likelihood descriptors were interpreted using the following scale: <0.5%, most likely; <5%, very unlikely; <25%, unlikely; 25–75%, possibly; >75%, likely; >95%, very likely; and >99.5%, most likely [28]. Additionally, all study variables were compared along the six-week training program using a *t*-test (pre- vs. post-1- vs. post-2), and the relationships between the changes from the laboratory and field tests during each training cycle were calculated with Pearson’s correlation coefficients. SPSS software version 25 (SPSS Inc., Chicago, IL, USA) was employed for this purpose. Data are presented as the mean ± standard deviation, and the statistical significance level was set at *p* < 0.05. 

## 3. Results

### 3.1. Effects of Flywheel Training from Pre- to Post-1 Conditions

**Figure 3:**
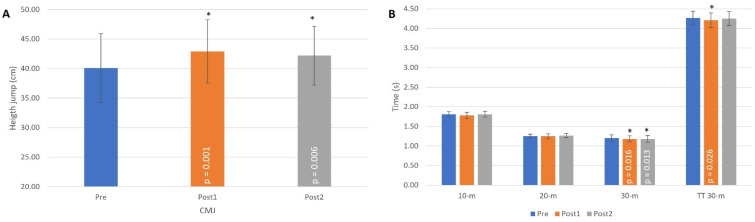

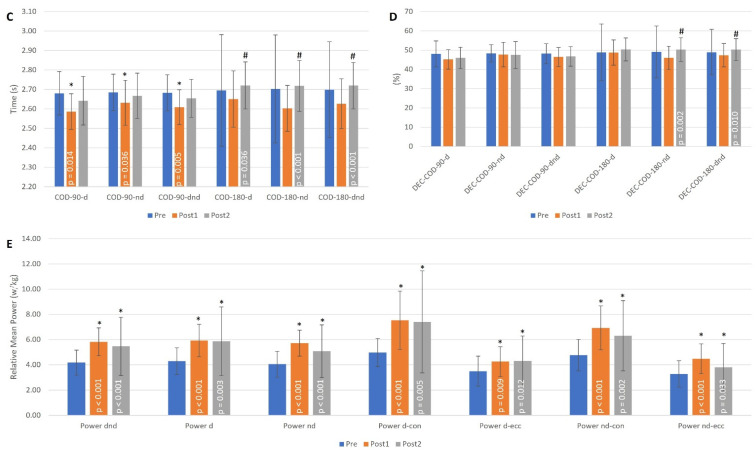
Evolution of CMJ (**A**), 30 m (**B**), COD (**C**), DEC-COD (**D**) and laboratory (**E**) variables during pre, post-1 and post-2 tests. (*) Different from the pre-test at *p* < 0.05; (#) Different from the post-1 test at *p* < 0.05. Abbreviations: CMJ, countermovement jump height; TT 30 m, 30 m total sprint time 30 m; COD, change of direction; DEC, percentage decrement respect 10 m; d, dominant leg; nd, non-dominant leg; dnd, mean value of both legs; con, concentric action; ecc, eccentric action.

The effects of flywheel training on the field and laboratory tests performed by sport science students between the pre- and the post-1 conditions are presented in Table 1. The CMJ, the 10 m, 30 m and TT 30 m sprint times, and all COD-90 tests (dominant and non-dominant) likely presented substantial beneficial effects after the first three weeks of the training program based on single-leg Romanian deadlifts on a flywheel device. Relationships between the total sprint time and COD-90 test likely decreased by the post-1 condition. In relation to the power tests, both eccentric and concentric tests with dominant and non-dominant legs presented a very likely large beneficial effect after three weeks of flywheel training. Statistical differences were obtained between pre- and post-1 tests in CMJ (Figure 3A), 30 m, TT 30 m (Figure 3B), COD-90-d, COD-90-nd, COD-90-dnd (Figure 3C), Power dnd (w/kg), Power d (w/kg), Power nd (w/kg), Power d-con (w/kg), Power d-ecc (w/kg), Power nd-con (w/kg), and Power nd-ecc (w/kg) tests (Figure 3E). The following relationships were found between the changes from the laboratory and field tests from pre- to post-1: moderate or large relationships between Power d with COD-180-d (r = −0.513; *p* = 0.035), COD-180-nd (r = −0.495; *p* = 0.043), and COD-180-dnd (r = −0.597; *p* = 0.011); and moderate relationships between Power d-con with CMJ (r = 0.49; *p* = 0.045), COD-180-d (r = −0.483; *p* = 0.05) and COD-180-dnd (r = −0.491; 0.045).

### 3.2. Effects of Flywheel Training from Post-1 to Post-2 Conditions

From the post-1 to the post-2 condition (Table 2), the field test showed likely detrimental effects of flywheel training in the COD-90 test with the dominant leg and in all COD-180 conditions, except with the dominant leg. Additionally, the relationship between the TT 30 m and the COD-180 test performance very likely increased. The power test, on the other hand, showed detrimental likely effects from post-1 to post-2 conditions in all tests favoring the dominant leg. Statistical differences were obtained between post-1 and post-2 tests in COD-180-d, COD-180-nd, COD-180-dnd (Figure 3C), DEC-COD-180-nd, and DEC-COD-180-dnd tests (Figure 3D). The following relationships were found between the changes produced in the laboratory and field tests from post-1 to post-2: large relationship between Power d-con with DEC-COD-90-dnd (r = 0.586; *p* = 0.017), as well as between Power nd-con with COD-180-dnd (r = −0.574; *p* = 0.020) and DEC-COD-90-d (r = 0.517; *p* = 0.040).

### 3.3. Effects of Flywheel Training from Pre- to Post-2 Conditions

Finally, the comparison between the pre- and the post-2 conditions (Table 3), showed likely beneficial effects of the flywheel training on the CMJ, 30 m, and COD-90 field tests. Additionally, performance in all the power tests in the post-2 condition presented at least very likely beneficial effects as compared with the pre-test performance. Statistical differences were obtained between pre- and post-2 tests in CMJ (Figure 3A), 30 m (Figure 3B), Power dnd (w/kg), Power d (w/kg), Power nd (w/kg), Power d-con (w/kg), Power d-ecc (w/kg), Power nd-con (w/kg), and Power nd-ecc (w/kg) tests (Figure 3E). The following relationships were found between the changes produced in the laboratory and field tests from pre- to post-2: large relationship between Power nd-con with 20 m sprint (r = 0.567; *p* = 0.022).

## 4. Discussion

The main aim of the present research was to analyze the flywheel training effects (two periods of three weeks) of single-leg Romanian deadlifts on several physical performance tests. Results indicated likely or very likely improvements in athletes on their jumping, sprinting, COD-90, and power abilities after the first three weeks of flywheel training. However, these improvements did not further improve in most field tests when the flywheel training volume was augmented after the initial 3 weeks.

### 4.1. Effects of the Flywheel Training from Pre- to Post-1 Condition

The jumping abilities of sport science students in the CMJ test presented very likely (7.3%) improvements after the first three weeks of intervention. This change was expected according to previous findings [9,29], but it was greater than that previously reported (between 4.4% and 4.7%) after longer protocols (5 to 7 weeks) of unilateral squat or lateral squat training in elite- or active-level athletes, respectively [7,14]. In relation to the sprinting abilities, 30 m total times likely improved by 1.4% after flywheel training, including a 1.6% improvement in the first 10 m and 0.6% in the 20–30 m segments. These results do not correspond to the effects of flywheel training with unilateral squat [14], where unclear effects on sprinting abilities to 10 m were observed; however, this equaled the effects of flywheel training observed over a whole season in young male professional soccer players [30]. Differences from the literature in both the jumping and sprinting effects could be related to the low inertia load employed in the present research as compared with the mentioned studies (0.037 kg/m^2^ in the present research compared with 0.05–0.10 kg/m^2^ used elsewhere). This could ease a greater velocity execution in the flywheel training of student athletes and, consequently, early adaptations in the muscle architecture based on a greater muscular fascicle length [31]. Indeed, it has been reported that muscle activation, as well as muscular stiffness, respond differently according to the inertia loads in flywheel training; therefore, they can be used for load prescriptions in flywheel devices [26,32]. Additionally, flywheel devices have been reported to stimulate faster increases in sarcomeres in series than in parallel, as compared with traditional systems, which would improve the velocity-related abilities [14].

For the 90° change of direction tests, times of student athletes improved from 2.0% to 3.5% depending on the non-dominant or dominant side, respectively. However, these changes were not observed in the COD-180 tests, where chances of a beneficial effect of flywheel training did not reach 60%. These results are in line with previously reported data [15], where similar improvements to in the COD-90 tests were observed. When comparing the sprint and the change of direction performance (DEC–COD), a decrement in differences was observed due to flywheel training (up to 6%), especially in the COD-90 test performed with the dominant limb. On the other hand, the pattern was the opposite with the COD-180 test, where differences in sprinting performance after flywheel training increased by up to 8.9% with the dominant limb. These changes are considerably lower than those presented elsewhere after unilateral squat training, where healthy active males showed DEC–COD decreases from 13% to 21% [14]. The reason could be a moderate to large correlation which has been observed between eccentric muscle strength levels and the COD performance [33], and which suggests that strength and power gains in the lower limbs could become more important in sharp turns and direction changes [34]. In this line, relationships observed in the present study between power gains and COD were greater than with linear running or jumping abilities. It should also be noted that unilateral squat training has been proposed to be more effective than bilateral squat training in improving performance in change of direction tests [14]. However, there is still no clear consensus on which training form is optimal to develop COD performance [35].

In relation to power tests, results from flywheel training after the first three weeks revealed beneficial effects both in the eccentric and concentric conditions (with dominant and non-dominant limbs), although the magnitudes of changes were greater for the concentric (around 40%) versus the eccentric conditions (maximum 24%). These power gains were similar to the results in bilateral squat [14] and slightly greater than reported after 5 weeks of flywheel training of the knee extensor muscles [36]. Additionally, differences observed between concentric and eccentric effects were in line with previous results [37], supporting the use of low inertial loads (≈0.025 to 0.05 kg/m^2^) to maximize the concentric effects. It should also be highlighted that the effects of flywheel training in the student athletes after three weeks were similar to previous protocols lasting a minimum of 5 weeks, which represents an important finding of the present research in order to facilitate implementation by athletes [19].

### 4.2. Effects of the Flywheel Training from Post-1 to Post-2 Condition

The evolution of the flywheel training responses after a prolonged training from week 4 to week 6 (post-2 condition) was considerably different from the initial period of the three-week training. Despite small beneficial effects which occurred in the dominant power tests, several conditions of the COD tests presented detrimental effects (up to 4% time increases) after this second period of training; unclear, or possibly no effects were observed in the remaining field tests (Table 2). This was observed despite the training load in the 4–6-week period being increased (from three to four sets per flywheel training session), as per previous protocols [10,34], although the results cannot easily be compared with other studies because no measurements from the middle of the flywheel training period have been reported. Our post-2 data indicate that the effects of the flywheel training on the student athletes seemed to occur on a short-term period, probably shorter than the 6 weeks of training employed elsewhere [38]. These short-term force gains with the flywheel device could be related to an increased recruitment and synchronization of motor units during exercise, in addition to an increase in EMG activity compared with traditional weight machines [39]. No muscle volume increases have been reported after flywheel training; longer training protocols would probably be required in order to see the effects [38]. Additional effects of flywheel training after the initial period of three weeks would probably require an adaptation period before increasing the training load, in accordance with general overload adaptation rules [2]. Specific changes observed in the power tests with the dominant limb in the post-2 condition, unlike with the non-dominant limb (Table 2), would confirm the different stimulation during flywheel training due to different inter-limb vertical ground forces [9], and it would have clear implications for training prescription and injury prevention programs [40,41].

### 4.3. Effects of the Flywheel Training from Pre- to Post-2 Condition

Finally, despite substantial effects of the flywheel training being observed primarily in the first three weeks of training, jump and power variables were maintained through the six weeks of flywheel training of student athletes (Table 3). This would indicate that prolonged flywheel training with an increased load after the initial three-week would maintain (but not improve) some of the training effects in student athletes. On the other hand, COD abilities of student athletes seemed to decline after the first three weeks of training (Figure 3C,D). The greater complexity of this skill, as well as no clear consensus on which training form is optimal to develop COD performance [35], justifies the need for further research on the load progression for COD performance. 

## 5. Conclusions

The single-leg Romanian deadlift training of student athletes with a flywheel device showed a likely substantial beneficial effect over the linear sprinting, jumping and COD performance, as well as power values during the first three weeks of training. On the other hand, likely detrimental effects were observed in some of the change of direction conditions in a second period of three weeks of training. In addition, jump and power variables were maintained throughout the complete six-week training period. It could be hypothesized that most of the flywheel training effects reported in the traditional protocols lasting a minimum of 5–6 weeks would occur in the first weeks of training.

### Practical Applications and Future Research

Using flywheel devices with a low moment of inertia and over a short-term period seems to provide greater effects on the jumping and linear sprinting abilities than previously reported protocols of a greater load. However, strength coaches should consider the dose-dependent responses of flywheel training. Training stimuli after three weeks or with an increased load after the initial period showed a clear decrease. Further research is needed to develop strategies that can maintain training stimulus and effects over an extended training period, as well as establish adequate progression in terms of periodization. The main limitations of the present research which must be acknowledged were the lack of a control group and information on the test protocol reliability. Additionally, changes in the muscle mass of athletes should be monitored to help explain training effects. 

## Figures and Tables

**Table 1:** Changes in performance after the first three-week period (pre- to post-1) of flywheel training.

Variables. Field Test	Pre Mean ± SD	Post-1 Mean ± SD	% Difference (90% CL)	Standardized Difference (90% CL)	Chances of Best/Trivial/Worse Effect	Qualitative Magnitude
CMJ (cm)	40.08 ± 5.84	42.89 ± 5.36	7.3 (4.7; 9.9)	0.46 (0.30; 0.62)	99/01/00	Very Likely
10 m (s)	1.81 ± 0.07	1.78 ± 0.08	−1.6 (−3.5; 0.4)	−0.42 (−0.94; 0.52)	03/21/77	Likely
20 m (s)	1.25 ± 0.05	1.25 ± 0.06	−0.6 (−2.0; 0.8)	−0.15 (−0.49; 0.20)	05/56/39	Unclear
30 m (s)	1.20 ± 0.08	1.18 ± 0.08	−0.6 (−2.0; −0.8)	−0.30 (−0.48; 0.12)	00/18/82	Likely
TT 30 m (s)	4.27 ± 0.17	4.21 ± 0.18	−1.4 (−2.4; −0.4)	−0.35 (−0.59; 0.10)	00/15/84	Likely
COD-90-d (s)	2.68 ± 0.11	2.59 ± 0.09	−3.5 (−5.5; −1.5)	−0.81 (−1.29;0.34)	00/02/98	Very Likely
COD-90-nd (s)	2.69 ± 0.09	2.63 ± 0.12	−2.0 (−3.6; −0.4)	−0.55 (−1.00;0.10)	01/09/90	Likely
COD-90-dnd	2.68 ± 0.09	2.61 ± 0.09	−2.7 (−4.2; −1.3)	−0.76 (−1.16;0.36)	00/01/99	Very Likely
COD-180-d (s)	2.70 ± 0.29	2.65 ± 0.14	−1.2 (−5.5; 3.2)	−0.10 (−0.46; 0.26)	08/60/31	Unclear
COD-180-nd (s)	2.70 ± 0.28	2.65 ± 0.12	−3.2 (−7.4; 1.1)	−0.27 (−0.63; 0.09)	02/35/61	Unclear
COD-180-dnd	2.70 ± 0.25	2.63 ± 0.13	−2.4 (−5.7; 1.1)	−0.24 (−0.59; 0.11)	02/40/58	Possibly
DEC-COD-90-d (%)	48.11 ± 6.72	45.20 ± 5.10	−5.8 (−12.0; 0.9)	−0.42 (−0.90; 0.07)	02/20/78	Likely
DEC-COD-90-nd (%)	48.33 ± 4.52	47.74 ± 6.38	−1.7 (−6.3; 3.3)	−0.18 (−0.69; 0.34)	11/42/47	Possibly
DEC-COD-90-dnd (%)	48.22 ± 5.16	46.47 ± 4.96	−3.6 (−8.0; 1.0)	−0.34 (−0.77; 0.09)	02/27/71	Possibly
DEC-COD-180-d (%)	48.81 ± 14.78	48.73 ± 6.53	8.9 (−13.0; 36.3)	0.15 (−0.24; 0.53)	41/53/07	Possibly
DEC-COD-180-nd (%)	49.14 ± 13.50	46.10 ± 6.04	1.6 (−19.1; 27.7)	0.03 (−0.36; 0.42)	23/61/16	Possibly
DEC-COD-180-dnd (%)	48.97 ± 11.85	47.41 ± 6.01	−0.3 (−11.8; 12.6)	−0.01 (−0.40; 0.38)	18/62/20	Possibly
Laboratory test						
Power dnd (w/kg)	4.18 ± 0.98	5.82 ± 1.11	40.0 (28.7; 52.2)	1.47 (1.10; 1.83)	100/00/00	Most Likely
Power d (w/kg)	4.30 ± 1.04	5.92 ± 1.29	38.2 (25.3; 52.4)	1.34 (0.93; 1.74)	100/00/00	Most Likely
Power nd (w/kg)	4.06 ± 1.02	5.72 ± 1.02	42.3 (30.2; 55.6)	1.46 (1.09; 1.83)	100/00/00	Most Likely
Power d-con (w/kg)	4.98 ± 1.10	7.53 ± 2.31	48.4 (31.8; 67.0)	1.75 (1.22; 2.27)	100/00/00	Most Likely
Power d-ecc (w/kg)	3.51 ± 1.19	4.26 ± 1.18	24.0 (6.6; 44.3)	0.57 (0.17; 0.97)	94/06/00	Likely
Power nd-con (w/kg)	4.77 ± 1.24	6.93 ± 1.75	44.8 (26.3; 66.1)	1.46 (0.92; 2)	100/00/00	Most Likely
Power nd-ecc (w/kg)	3.29 ± 1.03	4.48 ± 1.18	37.7 (20.7; 57.0)	1.04 (0.61; 1.47)	100/00/00	Most Likely

Abbreviations: CL, confidence limits; CMJ, countermovement jump height; TT, 30 m total sprint time 30 m; COD, change of direction; DEC, percentage decrement respect 10 m; d, dominant leg; nd, non-dominant leg; dnd, mean value of both legs; con, concentric action; ecc, eccentric action.

**Table 2:** Changes in performance after the second three-week period (post-1 to post-2) of flywheel training.

Variables. Field Test	Post-1, Mean ± SD	Post-2, Mean ± SD	% Difference (90% CL)	Standardized Difference (90% CL)	Chances of Best/Trivial/Worse Effect	Qualitative Magnitude
CMJ (cm)	42.89 ± 5.36	42.22 ± 4.98	−0.3 (−2.8; 2.2)	−0.02 (−0.18; 0.14)	02/95/04	Unclear
10 m (s)	1.78 ± 0.08	1.81 ± 0.0.08	1.3 (−0.2; 2.8)	0.34 (−0.04; 0.73)	74/25/01	Unclear
20 m (s)	1.25 ± 0.06	1.27 ± 0.0.05	1.1 (−0.3; 2.4)	0.26 (−0.06; 0.58)	62/36/01	Unclear
30 m (s)	1.18 ± 0.08	1.18 ± 0.09	−0.8 (−2.6; 1.1)	−0.11 (−0.38; 0.16)	03/69/28	Unclear
TT 30 m (s)	4.21 ± 0.18	4.25 ± 0.18	0.7 (−0.2; 1.5)	0.16 (−0.05; 0.37)	37/63/00	Unclear
COD-90-d (s)	2.59 ± 0.09	2.64 ± 0.12	1.7 (0.1; 3.3)	0.39 (−0.92; 0.08)	81/19/01	Likely
COD-90-nd (s)	2.63 ± 0.12	2.67 ± 0.12	1 (−1.7; 3.8)	0.27 (−0.47; 1.02)	57/29/14	Unclear
COD-90-dnd	2.61 ± 0.09	2.66 ± 0.10	1.4 (−0.4; 3.1)	0.37 (−0.10; 0.84)	73/24/03	Possibly
COD-180-d (s)	2.65 ± 0.14	2.72 ± 0.12	2.1 (0.6; 3.6)	0.17 (0.05; 0.28)	31/69/00	Unclear
COD-180-nd (s)	2.65 ± 0.12	2.72 ± 0.13	4.0 (2.5; 5.5)	0.32 (0.22; 0.44)	95/05/00	Very Likely
COD-180-dnd	2.63 ± 0.13	2.72 ± 0.12	3.0 (1.8; 4.2)	0.30 (0.18; 0.41)	91/09/00	Likely
DEC-COD-90-d (%)	45.20 ± 5.10	46.01 ± 5.51	1.1 (−4.8; 7.4)	0.08 (−0.34; 0.50)	31/56/13	Possibly
DEC-COD-90-nd (%)	47.74 ± 6.38	47.47 ± 6.98	−1.0 (−7.5; 5.9)	−0.11 (−0.83; 0.61)	23/36/41	Possibly
DEC-COD-90-dnd (%)	46.47 ± 4.96	46.74 ± 5.01	0.2 (−4.1; 4.7)	0.02 (−0.39; 0.42)	22/60/18	Possibly
DEC-COD-180-d (%)	48.73 ± 6.53	50.39 ± 5.92	2.3 (−1.8; 6.6)	0.04 (−0.3; 0.11)	00/100/00	Most Likely
DEC-COD-180-nd (%)	46.10 ± 6.04	50.24 ± 6.23	8.4 (4.6; 12.4)	0.14 (0.08; 0.20)	05/95/00	Very Likely
DEC-COD-180-dnd (%)	47.41 ± 6.01	50.31 ± 5.68	5.3 (2.2; 8.5)	0.16 (0.07; 0.26)	25/75/00	Possibly
Laboratory test						
Power dnd (w/kg)	5.82 ± 1.11	5.81 ± 1.88	9.6 (0.9; 19.0)	0.40 (0.04; 0.76)	83/17/01	Likely
Power d (w/kg)	5.92 ± 1.29	6.24 ± 2.33	14.0 (1.8; 27.7)	0.54 (0.07; 1.01)	89/10/01	Likely
Power nd (w/kg)	5.72 ± 1.02	5.40 ± 1.66	4.3 (−4.0; 13.2)	0.17 (−0.17; 0.51)	45/52/04	Possibly
Power d-con (w/kg)	7.53 ± 2.31	7.87 ± 3.68	11.0 (−2.7; 6.7)	0.46 (−0.12; 1.05)	78/19/03	Likely
Power d-ecc (w/kg)	4.26 ± 1.18	4.58 ± 1.69	18.9 (3.2; 37.0)	0.46 (0.08; 0.84)	88/12/00	Likely
Power nd-con (w/kg)	6.93 ± 1.75	6.70 ± 2.33	7.0 (−5.9; 21.7)	0.27 (−0.24; 0.77)	59/35/06	Possibly
Power nd-ecc (w/kg)	4.48 ± 1.18	4.05 ± 1.65	−0.8 (−12.8; 12.8)	−0.03 (−0.44; 0.39)	18/58/24	Possibly

Abbreviations: CL, confidence limits; CMJ, countermovement jump height; TT, 30 m total sprint time 30 m; COD, change of direction; DEC, percentage decrement respect 10 m; d, dominant leg; nd, non-dominant leg; dnd, mean value of both legs; con, concentric action; ecc, eccentric action.

**Table 3:** Changes in performance after two three-week periods (pre- to post-2) of flywheel training.

Variables. Field Test	Pre, Mean ± SD	Post-2, Mean ± SD	% Difference (90% CL)	Standardized Difference (90% CL)	Chances of Best/Trivial/Worse Effect	Qualitative Magnitude
CMJ (cm)	40.08 ± 5.84	42.22 ± 4.98	7.1 (3.3; 1.1)	0.45 (0.21; 0.69)	96/04/00	Very Likely
10 m (s)	1.81 ± 0.07	1.81 ± 0.08	−0.5 (−2.5; 1.5)	−0.14 (−0.67; 0.40)	14/44/42	Unclear
20 m (s)	1.25 ± 0.05	1.27 ± 0.05	0.5 (−0.9; 2.0)	0.13 (−0.22; 0.48)	37/57/06	Unclear
30 m (s)	1.20 ± 0.08	1.18 ± 0.09	−2.8 (−4.5; −1.1)	−0.41 (−0.66; −0.15)	00/09/91	Likely
TT 30 m (s)	4.27 ± 0.17	4.25 ± 0.18	−0.9 (−1.9; 0.2)	−0.21 (−0.46; −0.05)	01/48/51	Unclear
COD-90-d (s)	2.68 ± 0.11	2.64 ± 0.12	−1.8 (−4.0; 0.4)	−0.42 (−0.92; 0.08)	02/20/77	Likely
COD-90-nd (s)	2.69 ± 0.09	2.67 ± 0.12	−1.3 (−2.9; 0.4)	−0.35 (−0.79; 0.10)	02/26/71	Unclear
COD-90-dnd	2.68 ± 0.09	2.66 ± 0.10	−1.5 (−3.0; 0.0)	−0.42 (−0.84; 0.00)	01/18/81	Likely
COD-180-d (s)	2.70 ± 0.29	2.72 ± 0.12	1.0 (−3.7; 6.1)	0.08 (−0.31; 0.48)	31/58/11	Unclear
COD-180-nd (s)	2.70 ± 0.28	2.72 ± 0.13	0.6 (−3.9; 5.3)	0.05 (−0.33; 0.43)	25/62/13	Unclear
COD-180-dnd	2.70 ± 0.25	2.72 ± 0.12	0.7 (−3.0; 4.4)	0.7 (−0.30; 0.43)	27/62/11	Possibly
DEC-COD-90-d (%)	48.11 ± 6.72	46.01 ± 5.51	−3.98 (−10.3; 2.9)	−0.28 (−0.76; 0.20)	05/34/61	Possibly
DEC-COD-90-nd (%)	48.33 ± 4.52	47.47 ± 6.98	−2.8 (−8.5; 3.2)	−0.30 (−0.93; 0.33)	09/30/61	Possibly
DEC-COD-90-dnd (%)	48.22 ± 5.16	46.74 ± 5.01	−3.1 (−7.9; 1.9)	−0.29 (−0.76; 0.17)	04/32/63	Possibly
DEC-COD-180-d (%)	48.81 ± 14.78	50.39 ± 5.92	13.5 (−11.5; 45.5)	0.22 (−0.21; 0.64)	53/42/05	Possibly
DEC-COD-180-nd (%)	49.14 ± 13.50	50.24 ± 6.23	11.5 (−11.5; 40.5)	0.19 (−0.21; 0.58)	48/47/05	Possibly
DEC-COD-180-dnd (%)	48.97 ± 11.85	50.31 ± 5.68	6.2 (−6.6; 20.6)	0.19 (−0.22; 0.60)	48/46/06	Possibly
Laboratory test						
Power dnd (w/kg)	4.18 ± 0.98	5.81 ± 1.88	53.4 (39.1; 69.2)	1.87 (1.44; 2.30)	100/00/00	Most Likely
Power d (w/kg)	4.30 ± 1.04	6.24 ± 2.33	58.9 (38.3; 82.6)	1.91 (1.34; 2.49)	100/00/00	Most Likely
Power nd (w/kg)	4.06 ± 1.02	5.40 ± 1.66	47.1 (34.3; 61.2)	1.60 (1.22; 1.97)	100/00/00	Most Likely
Power d-con (w/kg)	4.98 ± 1.10	7.87 ± 3.68	66.8 (38.3; 101.2)	2.26 (1.44; 3.09)	100/00/00	Most Likely
Power d-ecc (w/kg)	3.51 ± 1.19	4.58 ± 1.69	48.4 (27.3; 73.0)	1.05 (0.64; 1.45)	100/00/00	Most Likely
Power nd-con (w/kg)	4.77 ± 1.24	6.70 ± 2.33	53.9 (34.3; 76.5)	1.70 (1.16; 2.23)	100/00/00	Most Likely
Power nd-ecc (w/kg)	3.29 ± 1.03	4.05 ± 1.65	34.8 (14.8; 58.4)	0.97 (0.45; 1.49)	99/01/00	Very Likely

Abbreviations: CL, confidence limits; CMJ, countermovement jump height; TT, 30 m total sprint time 30 m; COD, change of direction; DEC, percentage decrement respect 10 m; d, dominant leg; nd, non-dominant leg; dnd, mean value of both legs; con, concentric action; ecc, eccentric action.

## Data Availability

The data presented in this study are available on request from the corresponding author. The data are not publicly available due to restrictions privacy.
